# Spin-dependent transport and functional design in organic ferromagnetic devices

**DOI:** 10.3762/bjnano.8.192

**Published:** 2017-09-13

**Authors:** Guichao Hu, Shijie Xie, Chuankui Wang, Carsten Timm

**Affiliations:** 1 School of Physics and Electronics, Shandong Normal University, Jinan 250014, China; 2 Institute of Theoretical Physics, Technische Universität Dresden, 01062 Dresden, Germany; 3 School of Physics, Shandong University, Jinan 250100, China

**Keywords:** magnetoresistance, organic ferromagnet, spin-current rectification, spin filtering, transport

## Abstract

Organic ferromagnets are intriguing materials in that they combine ferromagnetic and organic properties. Although challenges in their synthesis still remain, the development of organic spintronics has triggered strong interest in high-performance organic ferromagnetic devices. This review first introduces our theory for spin-dependent electron transport through organic ferromagnetic devices, which combines an extended Su–Schrieffer–Heeger model with the Green’s function method. The effects of the intrinsic interactions in the organic ferromagnets, including strong electron–lattice interaction and spin–spin correlation between π-electrons and radicals, are highlighted. Several interesting functional designs of organic ferromagnetic devices are discussed, specifically the concepts of a spin filter, multi-state magnetoresistance, and spin-current rectification. The mechanism of each phenomenon is explained by transmission and orbital analysis. These works show that organic ferromagnets are promising components for spintronic devices that deserve to be designed and examined in future experiments.

## Introduction

In recent years, organic spintronics has attracted more and more interest [1–3] in order to develop cheap and flexible devices employing the electronic spin degree of freedom. Organic spintronics has several merits compared with inorganic materials. The spin–orbit and hyperfine interactions in organic materials are usually weak [4], which induces a long spin relaxation time and makes organic materials ideal for spin-polarized transport applications. Organic molecules may form a soft interface with metals and ferromagnets via chemical adsorption. The interfacial orbital hybridization may modify the organic interfacial spin polarization (SP), which has triggered the new concept of “organic spinterface” [5]. Recent experimental studies have demonstrated the reproduction of conventional spintronic devices using organic counterparts, e.g., magnetoresitive devices [6–8]. On the other hand, the search for novel functional organic materials remains of high interest for theorists and experimentalists.

Organic ferromagnets (OFs), which combine ferromagnetic and organic properties, are particularly promising for the design of organic spintronic devices. OFs are mainly synthesized artificially, such as by doping transition-metal ions into organic materials or using spin radicals [9–13]. The latter method may generate pure OFs. For example, poly((1,4-bis(2,2,6,6-tetramethyl-4-hydroxy-4-piperidyl-1-oxyl)butadiyne) (poly-BIPO) is a representative of π-conjugated pure OFs with quasi-one-dimensional structure, which can be synthesized from polyacetylene by replacing every other H atom by a spin radical. The radicals are usually heterocycles containing an unpaired electron. The spins of the radicals are coupled to the spins of π-electrons in the main carbon chain. Theoretical studies have found that the radical spins are ferromagnetically ordered in the ground state [14–15]. The magnetic properties of poly-BIPO have been measured experimentally, including high Curie temperature (150–190 °C) [9], magnetic hysteresis with residual magnetization (0.025–0.05 emu/g), and coercive force (295–470 Oe) [10], which are promising for spintronic applications. Although the chemical instability of the radicals still remains a challenge, remarkable progress has recently been made, where several classes of stable spin radicals have been obtained [9–10,16].

In the past decades, the research on OFs has focused on the synthesis, measurement, and characterization of the magnetic properties of the isolated molecules. Recently, the progress in organic and molecular spintronics has motivated us to explore the spin-dependent transport properties of OFs and the possibility to design organic ferromagnetic devices. A related field involves single molecular magnets (SMMs). Extensive experimental and theoretical studies have demonstrated the realization of functional devices based on SMMs, such as molecular switches and negative differential resistance [17–24]. Electronic transport in organic magnets has also been studied. Yoo et al. [25] have experimentally demonstrated the magnetic response of V[TCNE]*_x_*-based devices (TCNE stands for tetracyanoethylene) by connecting the organic magnet to gold electrodes. Li et al. [26] have investigated the magnetoresistance effect in organic magnetic devices with one ferromagnetic and one nonmagnetic electrode. Wang et al. [27–28] have performed theoretical studies of electron transport in OFs and have proposed spin–charge separation and spin filtering.

π-Conjugated OFs with spin radicals are ideal materials for device design since the π-orbitals are available for electron transport. The pursuit of novel effects based on the intrinsic properties of the OFs is one of our aims. Prior to that, the role of the following interactions needs to be clarified: First, the coupling between the spins of conducting π-electrons and the radical spins is the origin of the magnetism. How does it affect the spin-dependent transport? Second, the electron–lattice (e–l) interaction is strong in organic materials, which leads to dimerization in the ground state and opens a Peierls gap in the molecular energy band [29]. What is the role of the e–l interaction for the spin-dependent electron transport? Third, in the presence of the above two interactions, how do the molecular π-orbitals respond to an external bias? What is its manifestation in the current?

In the remainder of the paper, we introduce our theory for the electron transport through OF devices, which combines the extended Su–Schrieffer–Heeger (SSH) model [30] and the Green’s function method. The two interactions mentioned above are included. Then, we review results on electron transport and functional design of organic ferromagnetic devices, which are based on this theory. We focus on three concepts that are interesting for spintronics, namely spin filtering [31], multi-state magnetoresistance [32], and spin–current rectification [33].

## Review

### SSH model combined with the Green’s function method

**Figure 1:**
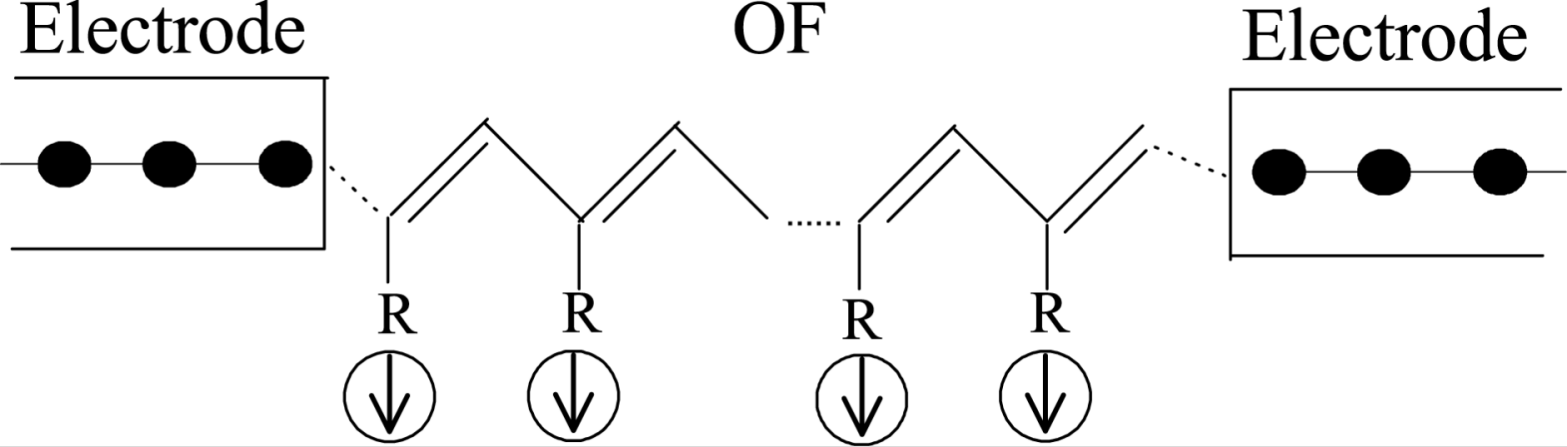
Schematic of an organic ferromagnetic device.

Generally, an OF device may be constructed by sandwiching the OF molecule between two electrodes, as shown in Figure 1. The two semi-infinite one-dimensional electrodes may be ferromagnetic or nonmagnetic metals. The central OF, such as poly-BIPO, consists of the main carbon chain and spin radicals attached to the odd sites. The OF can be described by an extended SSH model [30] combined with a Kondo term, which captures both the strong e–l interaction and the spin correlation between π-electrons and spin radicals. The Hamiltonian is written as [14–15]

[1]
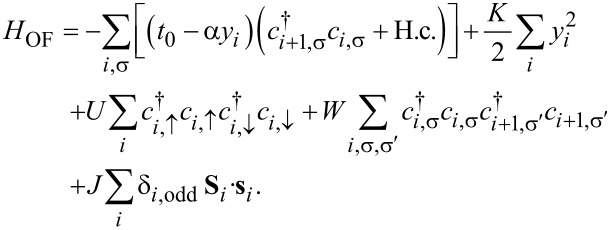


The first two terms are the expression of the SSH model [30] for an organic molecular chain. The former one is the hopping term of π-electrons along the main chain, where the hopping integral is modulated by the possible lattice distortion. *t*_0_ is the nearest-neighbor hopping integral in a uniform chain. α denotes the e–l coupling constant. *y_i_* corresponds to the lattice distortion *y_i_*≡ *u_i+1_* − *u_i_*, where *u_i_* is the displacement of the carbon atom at site *i*. 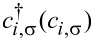 is the creation (annihilation) operator of an electron at site *i* with spin σ. The second term is the classical elastic energy of the lattice atoms in the main chain, where *K* is the elastic coefficient. The third and forth terms are the electron–electron interactions between π-electrons, where *U* and *W* are the on-site and nearest-neighbor interaction strengths, respectively. The last term is the spin coupling between the π-electron spins 
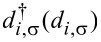
 and the radical spins **S***_i_*, with strength *J >* 0. This term contains δ*_i_*_,odd_ = 1 (δ*_i_*_,odd_ = 0) for *i* odd (even), which ensures that the spin coupling only occurs for the odd sites.

Since we focus on the effects of the interactions in the organic ferromagnet on transport, we model the electrodes by simple one-dimensional chains described by a single-band tight-binding model with a spin-splitting term [34],

[2]
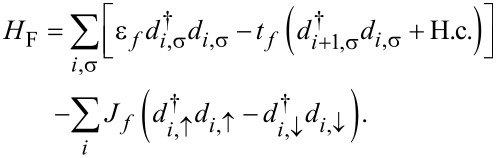


Here, 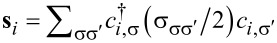 denotes the creation (annihilation) operator of an electron in the metal at site *i* with spin σ. ε*_f_* is the on-site energy of a metallic atom and *t_f_* the nearest-neighbor hopping integral. *J_f_* is a Stoner-type exchange field, which is set to zero for a nonmagnetic metal. The coupling between the electrode and the OF is described by a transfer integral *t_mf_*. Since our focus is on effects coming from the bulk of the OF chain, we here assume spin-independent coupling between the OF and the electrodes.

When a bias voltage *V* is applied, a spatially varying electric potential is generated along the molecule, which modifies both the electronic and the lattice structure. If the bias is not too large, a linear treatment is justified [35–36], where we assume that a uniform electric field *E* = *V*/[*a*(*N* − 1)] along the molecule is induced. Here, *N* is the total number of carbon atoms in the main chain and *a* is the lattice constant. Hence, the Hamiltonian involving the electric potential is

[3]
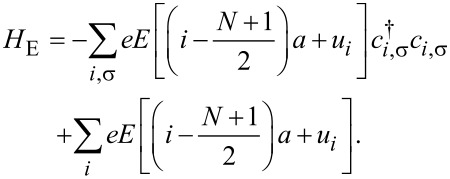


Here, *e* is the electronic charge of an electron. The first term is the electric potential of the π-electrons, and the second term is the potential of the ion cores.

Before calculating the transport properties, one needs to obtain the stationary structure of the OF under bias. Using the mean-field approximation to treat the electron–electron and spin–spin interactions, the eigenenergies ε_μ_*_,_*_σ_ and the eigenstates 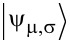 with (real) eigenfunctions ψ*_μ,σ,i_* can be calculated in Wannier space by solving the Schrödinger equation

[4]
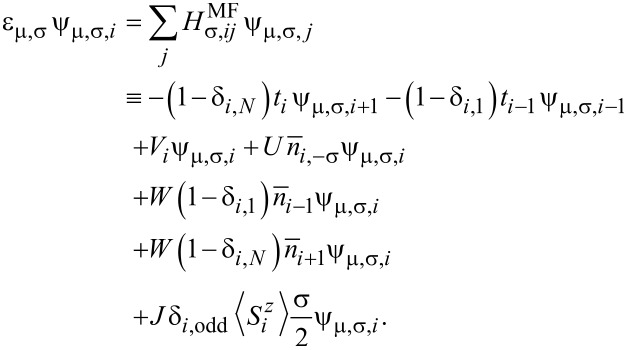


Here, we set 
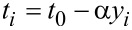
 and


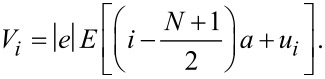
.


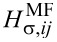
 is the matrix element of the mean-field Hamiltonian for the π-electrons with spin σ. The spin quantum number σ assumes the numerical values ↑ ≡ 1 and ↓ ≡ −1. 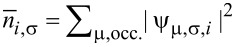 is the average occupation number of π-electrons at site *i* with spin σ. The sum is over all occupied states. 
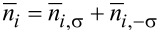
 is the total occupation number at site *i*. 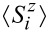 is the average value of the radical spin, assumed to be in the *z*-direction. The lattice distortion *y_i_* in Equation 4 is determined by minimizing the total energy, ∂*E*({*u_i_*})/∂{*u_i_*} = 0, which leads to the equation

[5]
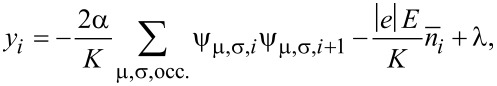


where the Lagrange multiplier λ guarantees that the length of the molecular chain remains unchanged, i.e., 
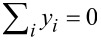
. A fixed-end boundary condition is adopted since the two ends of the molecular chain are attached to the electrodes. Equation 4 and Equation 5 are solved self-consistently [14].

In the regime of coherent transport, the current with spin σ through the device can be calculated from the Landauer-Büttiker formula [37]

[6]
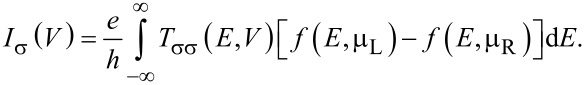


Here, 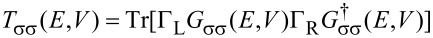 is the spin-dependent transmission coefficient determined from the retarded single-particle Green’s function *G*_σσ_(*E*,*V*) for the central OF [38]. Γ_L/R_ denotes the broadening matrix and *f*(*E*,μ_L/R_) is the Fermi distribution function of the left (L) or right (R) electrode with electrochemical potential μ_L/R_ = *E*_F_ ± eV/2 and Fermi energy *E*_F_.

For the numerical calculations we use parameter values appropriate for poly-BIPO [14,31,39]: *t*_0_ = 2.5 eV, α = 4.1 eV/Å, *K* = 21.0 eV/Å^2^, and 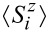 = −1/2. We introduce dimensionless interaction strengths *j* = *J*/*t*_0_, *u* = *U*/*t*_0_, and *w* = *W*/*t*_0_. The parameters for the electrodes vary according to the material adopted. For details on the parameters, see the related works [31–33].

### Spin filtering in metal/OF/metal devices

A spin filter is meant to generate a strongly spin-polarized current from an unpolarized current source and is a crucial element for spintronics. Using a magnetic interlayer in a sandwich structure is a valid method that has been reported in inorganic devices, such as Ag/EuSe/Al and Ag/EuS/Al [40–41]. To obtain a current with strong SP in those devices, a strong magnetic field is usually necessary to generate either spin-selective barriers or spin splitting of the resonant level. Interlayers made of OFs deserve to be explored for the possibility to realize an intrinsic organic spin filter. Here, we review progress in this direction made by some of us [31].

**Figure 2:**
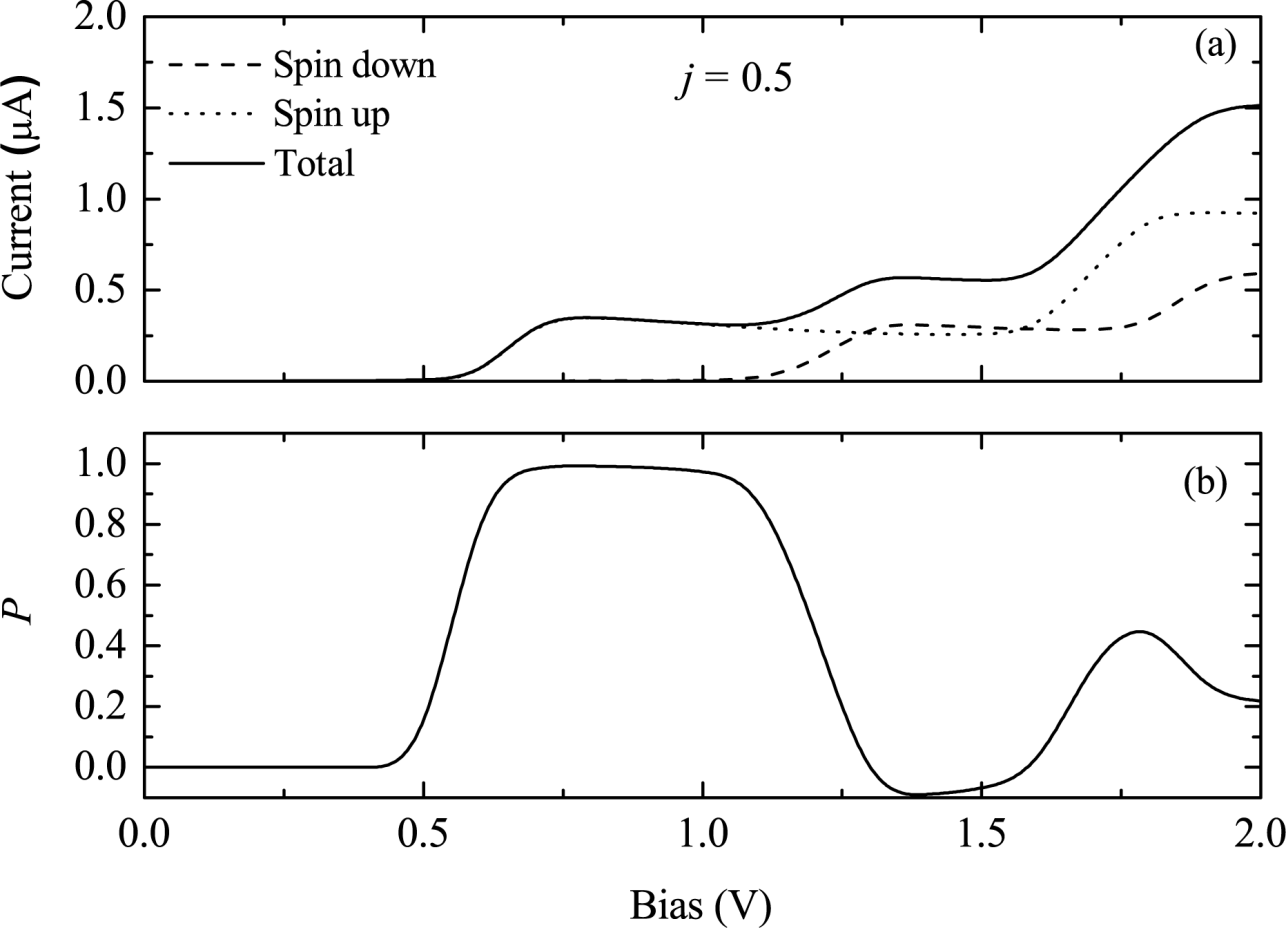
(a) Current–voltage characteristics for a OF device with *N* = 20 carbon sites. (b) Spin polarization of the current as a function of bias. Reproduced with permission from [31], copyright 2007 American Physical Society.

We have constructed the OF device by sandwiching the OF molecule between two identical nonmagnetic electrodes [31]. The spin-resolved and the total current calculated using the theory discussed in the previous section are shown in Figure 2a. The SP *P* = (*I*_↑_ − *I*_↓_)/(*I*_↑_ + *I*_↓_) of the current is given in Figure 2b. We have found a step-like current–voltage curve with a threshold voltage, which is common in nanojunctions. The spin-up and spin-down currents differ both in threshold voltage and magnitude. This leads to a non-monotonic dependence of the SP on bias, as shown in Figure 2b. In particular, nearly complete SP is obtained in the bias range of [0.7, 1.0] V, which means that strong spin filtering is realized in this bias range. The second peak of the SP appears at about 1.8 V but the SP is reduced to about 40%.

**Figure 3:**
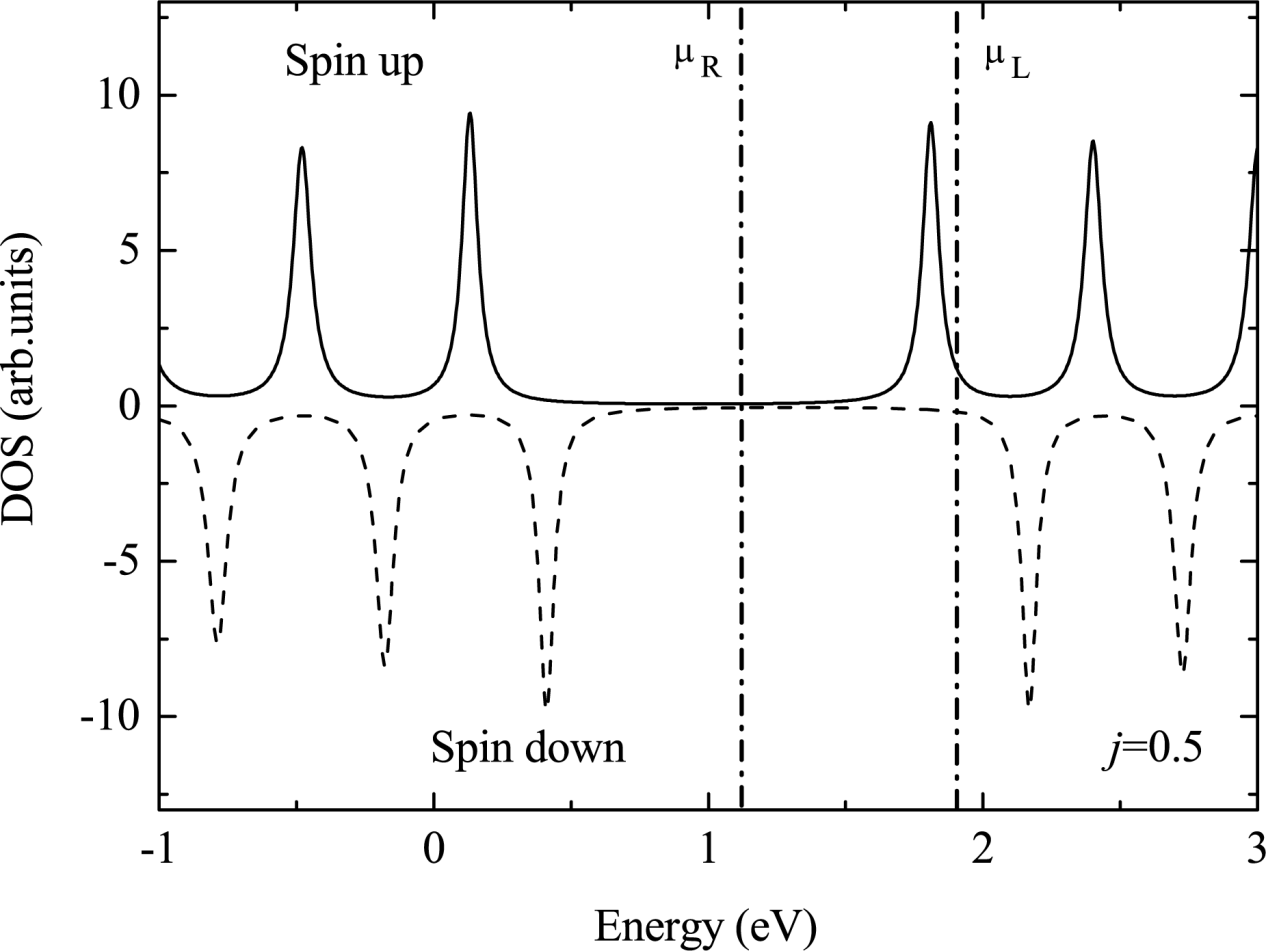
Density of states of the OF device at a bias of 0.8 V. Here, the Fermi energy of the electrodes is taken to be *E*_F_ = 1.5 eV, which for a bias voltage of *V* = 0.8 V leads to the indicated chemical potentials μ_L,R_ = *E*_F_ ± 0.4 eV. Reproduced with permission from [31], copyright 2007 American Physical Society.

In order to understand the spin filtering effect, we have calculated the spin-resolved density of states (DOS) of π-orbitals from the Green’s function with DOS_σ_(*E*,*V*) = −(1/π)Im[*G*_σσ_(*E*,*V*)]. The result for 0.8 V is shown in Figure 3. Evidently, the DOS is spin-split due to the coupling with radical spins. An energy gap of about 1.0 eV appears between the spin-down highest occupied molecular orbital (HOMO) and the spin-up lowest occupied molecular orbital (LUMO). For a bias of 0.8 V, only the spin-up LUMO falls into the bias window and contributes to the current. Therefore, the current is nearly fully spin polarized. When the bias increases, additional spin-up and spin-down orbitals will enter the bias window alternately, which results in the oscillation of the SP. Full spin filtering will not be reached again since the current includes contributions from electrons with different spins.

**Figure 4:**
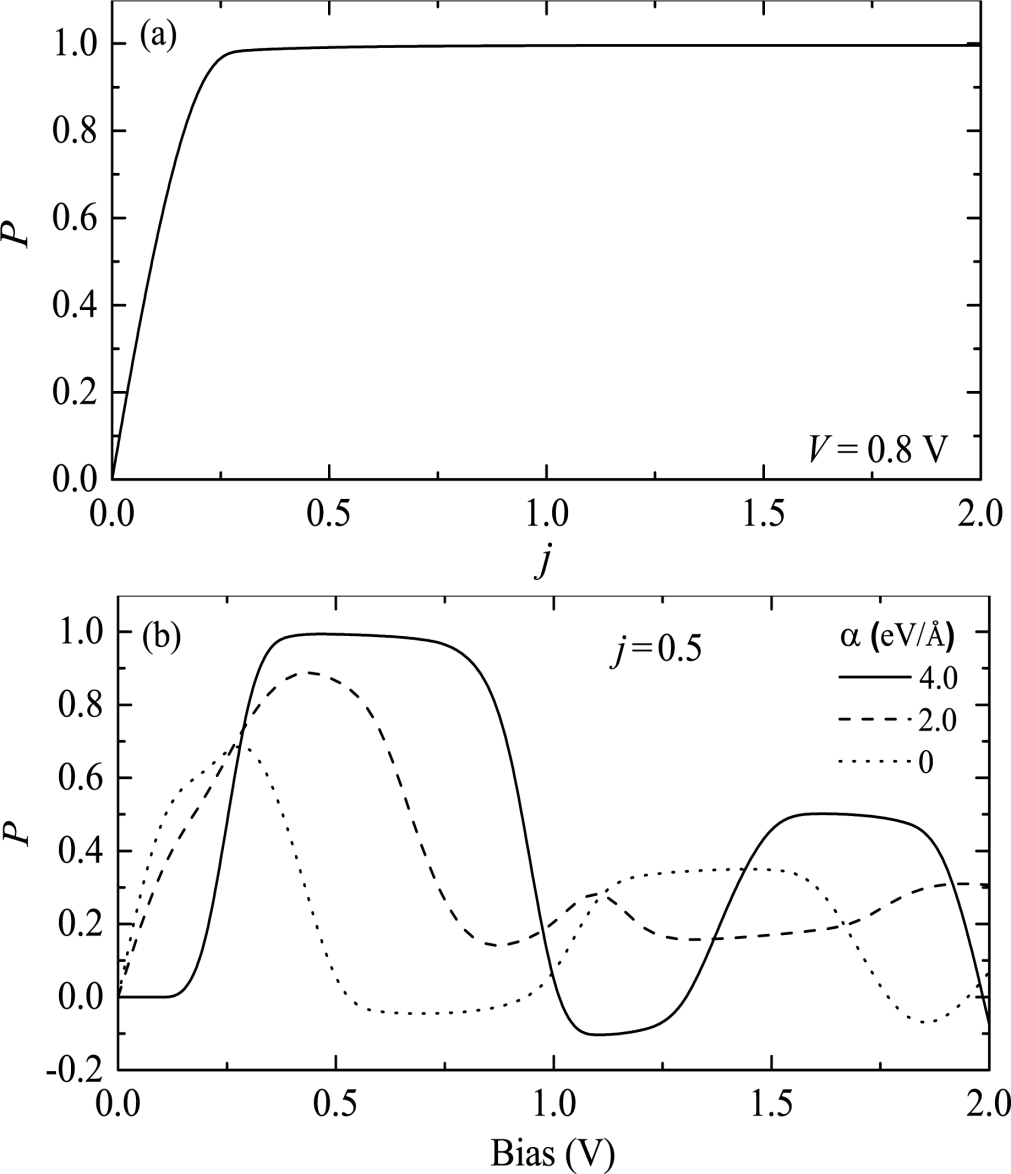
(a) Spin polarization of the current as a function of the spin coupling strength *j* for a bias of 0.8 V. (b) Bias-dependent spin polarization of the current for three different values of the electron–lattice coupling strength α. Reproduced with permission from [31], copyright 2007 American Physical Society.

The effects of the spin–spin and e–l interactions on the SP need to be clarified. The dependence of the SP on the spin–spin correlation parameter *j* is shown in Figure 4a, where the bias is fixed to 0.8 V. It is found that the SP increases rapidly, and then reaches the maximum value of nearly 100% at about *j* = 0.25. The intrinsic mechanism can be understood as follows: Without spin coupling, the molecular π-orbitals are spin-degenerate and the SP of the current is zero. In the case of nonzero *j*, a spin splitting of the π-orbitals occurs, where the spin-up orbitals are lowered in energy and the spin-down orbitals are raised. This spin splitting reduces the number of spin-down states in the conducting bias window, while it increases the number of spin-up states. As a result, the SP is increased. When the spin-down states are completely pushed out of the bias window, a nearly complete SP is achieved. Note that due to the existence of a large Peierls gap arising from the e–l interaction, the bias window continues to contain only spin-up states when *j* is increased further so that the SP will remain close to 100%. This means that the strong e–l interaction is crucial for spin filtering.

To elucidate the role of e–l interaction, we show, in Figure 4b, the SP of the current for three different strengths α of the e–l interaction at *j* = 0.5. α = 0 corresponds to a rigid chain without dimerization. In the case of a vanishing (α = 0) or weak (α = 2.0) e–l interaction, the SP occurs as soon as the bias is applied. However, for a strong e–l interaction (α = 4.0), a threshold voltage of about 0.2 V appears. Moreover, the maximum SP and the plateau width at the maximum SP increase with the e–l interaction. The reason is explained in the following: The spin coupling *j* induces a spin splitting of the π-orbitals, while the e–l interaction generates a Peierls gap separately for the orbitals with different spins. With the present parameters, the Peierls gaps for different spins are not symmetric to the Fermi level of the electrode. The numbers of spin-up and spin-down orbitals falling into the bias window are different, which is adjusted by the e–l interaction. This is the reason why the SP depends on the e–l interaction. It is noted that a nearly complete SP is obtained only for strong e–l interaction. In our calculation, there is a Peierls gap of about 1.65 eV. When the bias is increased to 0.3 V, only one spin-up orbital falls into the bias window, whereas the spin-down DOS is very small due to the Peierls gap. This leads to nearly complete SP. In the case of zero or weak e–l interaction, both spin-up and spin-down orbitals are close to the Fermi energy and contribute to the current, which is thus not fully spin polarized. An analogous phenomenon has been reported in a double-bend structure of a quantum wire, where an antiresonance gap is generated by weak lateral magnetic modulations, which leads to a large SP of the current [42]. Wang [27] also proposed a spin filtering effect in the same material coupled to a quantum wire, which is assumed to be manipulated by a gate voltage. We note that although a different model without e–l interaction was adopted in his work, a Hubbard gap still appears in the molecular band to separate the spin-up and spin-down energy levels.

The spin filtering effect in OF devices was also reported in other theoretical works. For the SSH model and using a Green’s function method, Sadaghi et al. [43] have investigated the spin-dependent transport through an OF chain with an odd number of sites, where a soliton in the main chain preexists. They found that spin filtering takes place when the spins of the soliton and the radicals point in opposite directions. Sun [44] has discussed the SP of the current through OF devices in the presence of ferromagnetic leads. A large SP is obtained in a specific bias region, which is enhanced by the polarization ratio of the magnetic electrode, and suppressed by the on-site Coulomb repulsion. Even in a long OF polymer chain, a spin filtering effect was obtained in the regime of polaron transport. Wang et al. [45] have found that a polaron moving under an electric field may be trapped near the spin radicals unless the field is stronger than a critical value. The magnitude of the critical field depends on the spin of the polaron, which implies a spin-filtering effect of the polaron transport.

### Multi-state magnetoresistance in ferromagnet/OF/ferromagnet junctions

Further control over an OF device can be gained by employing ferromagnetic electrodes. Ferromagnetic junctions are the basic building blocks for spin valves to realize the magnetoresistance (MR) effect, which is important in spintronics for magnetic storage. By manipulating the relative magnetic magnetization of the two electrodes with a magnetic field, e.g., parallel or antiparallel, the resistance of the device can be switched between low-resistance and high-resistance states. The utilization of organic molecules as the interlayer has been studied in many experiments, motivated by the long spin relaxation time [4]. Examples are the giant magnetoresistance (GMR) and the room-temperature tunneling magnetoresistance (TMR) in LSMO/Alq_3_/Co junctions [6–8].

**Figure 5:**
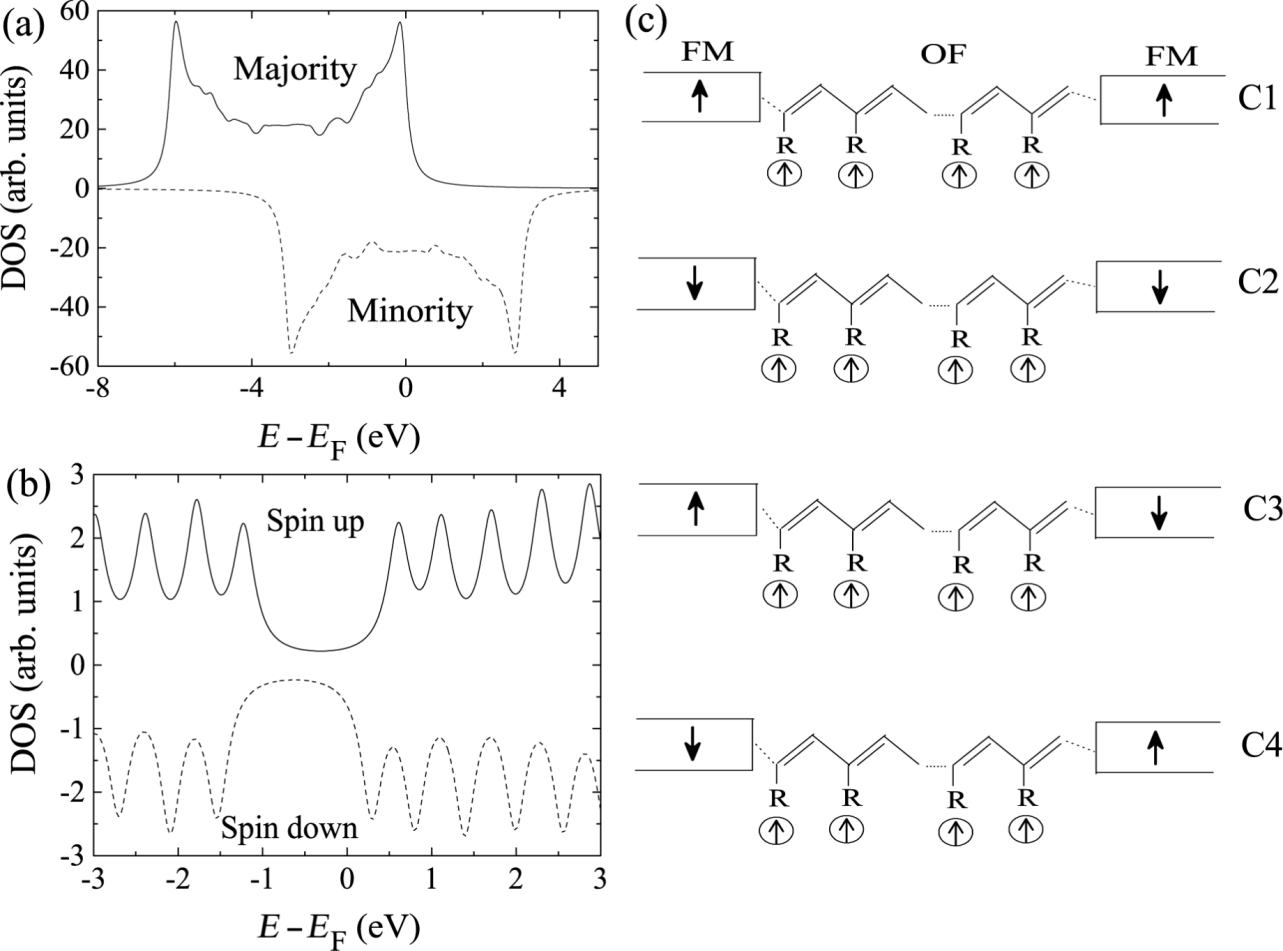
Density of states of (a) Co and (b) the OF poly-BIPO. The molecular length is 20 sites. (c) Schematic of the magnetization configurations C1, C2, C3, and C4 in the ferromagnet/OF/ferromagnet device. Reproduced with permission from [32], copyright 2014 AIP Publishing.

The MR in the ferromagnet/OF/ferromagnet junction Co/poly-BIPO/Co has been studied theoretically in the work [32], which we here review. The DOS of the isolated Co electrode and the OF are shown in Figure 5a and Figure 5b, respectively. The DOS of Co is consistent with one fully occupied majority-spin band and one half-filled minority-spin band [46]. Because all three components of the device are ferromagnetic, there exist four fundamentally distinct collinear alignments of the magnetizations. We fix the radical spins of the OF in the *z*-direction, while the magnetization of each ferromagnet may be parallel or antiparallel to the *z*-direction. The four configurations are labeled as C1 (↑↑↑), C2 (↓↑↓), C3 (↑↑↓), and C4 (↓↑↑) and are illustrated in Figure 5c.

**Figure 6:**
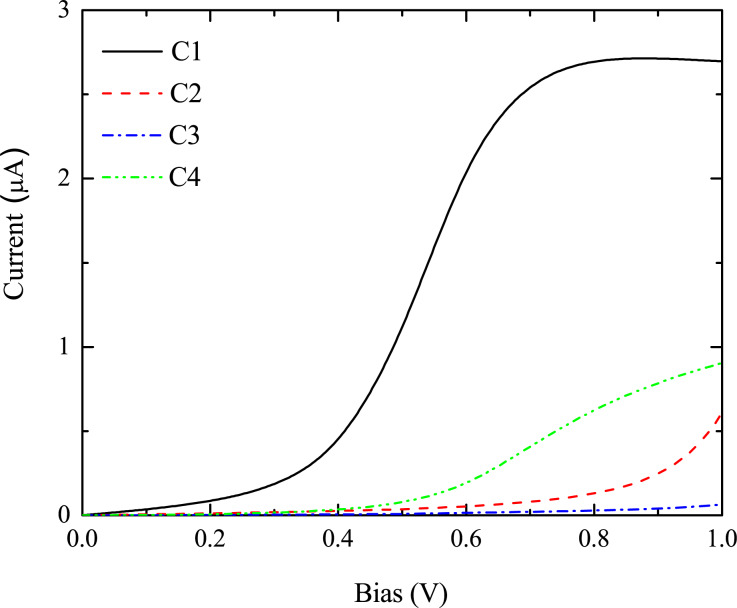
Current–voltage characteristics of a Co/OF/Co junction for the four magnetization configurations C1, C2, C3, and C4 shown in Figure 5c. Reproduced with permission from [32], copyright 2014 AIP Publishing.

The current–voltage characteristics for the four magnetization configurations are shown in Figure 6. It is found that the threshold voltage and the maximum magnitude of the current strongly depend on the magnetization configuration. Configuration C1 conducts first, with the smallest threshold voltage of about 0.3V. The maximum current of about 2.7 μA is reached when the bias exceeds 0.8 V. Larger threshold voltages of about 0.9 V and 0.6 V, respectively, is observed for C2 and C4. The current at 1.0 V is 0.5 μA for C2 and 0.9 μA for C4. On the other hand, the current for C3 is strongly suppressed within the calculated bias region. The different transport properties indicate that a multi-state MR effect can be realized by controlling the magnetization orientations of the electrodes and the central OF. One can quantify the bias-dependent multi-state MR as MR*_i_*(*V*) = [*R*_C_*_i_*(*V*) − *R*_C1_(*V*)]/*R*_C_*_i_*(*V*). Threshold voltage, maximum current, and multi-state MR for each case are summarized in Table 1. Obviously, four values of MR are realized with the change of the magnetization configuration. A maximum MR of 98% is obtained at a bias of 1.0 V.

**Table 1:** Threshold voltage (*V*_th_), maximum current for bias voltage in [0,1] V (*I*_max_), and multi-state magnetoresistance (MR) at a bias voltage of 1 V for the different magnetization configurations. Reproduced with permission from [32], copyright 2014 AIP Publishing.

configuration	*V*_th_ (V)	*I*_max_ (μA)	MR_i_ (*V* = 1.0 V)

C1 (↑↑↑)	0.3	2.7	0
C2 (↓↑↓)	0.9	0.5	82%
C3 (↑↑↓)	*>*1.0	0.06	98%
C4 (↓↑↑)	0.6	0.9	66%

The mechanism of the multi-state MR can be understood as follows: In the present device, electrons tunnel between the Co electrodes through the OF interlayer. In the two-current model [47], and according to the band structure of Co, the electron tunneling in C1 (C2) happens between the two half-filled spin-down (spin-up) Co bands. The situation is different in C3 (C4), where the tunneling takes place from the completely filled spin-up (spin-down) band of the left electrode to the half-filled spin-up (spin-down) bands of the right electrode. This difference is the origin of TMR in conventional spin valves. If the central layer is nonmagnetic, the resistance for C1 should be same as the one for C2, and analogously for C3 and C4. Thus two-state MR, for parallel and antiparallel alignments, will be obtained. However, in the presence of the OF, the π-orbitals in the OF are spin-split. In particular, spin filtering occurs near the Fermi energy, as discussed in the previous section on spin filtering. So, the spin-dependent tunneling for parallel and antiparallel configurations will suffer a further spin selection in the OF, which will induce a splitting of the resistance depending on the spin of the transported electrons. There is a pronounced difference between the currents for configurations C3 and C4, the magnetization configurations of which are mirror images of one another. The main origin of this asymmetry is that in the right (drain) electrode the spin-down band is completely occupied for C3 so that spin-down electrons cannot tunnel into it; spin-down electrons carry most of the current, as shown below. On the other hand, for C4 the spin-down band in the drain electrode is half filled. The additional asymmetry due to the spin radicals being attached only to odd sites, while the length of the OF is even (*N* = 20), does not play a large role.

**Figure 7:**
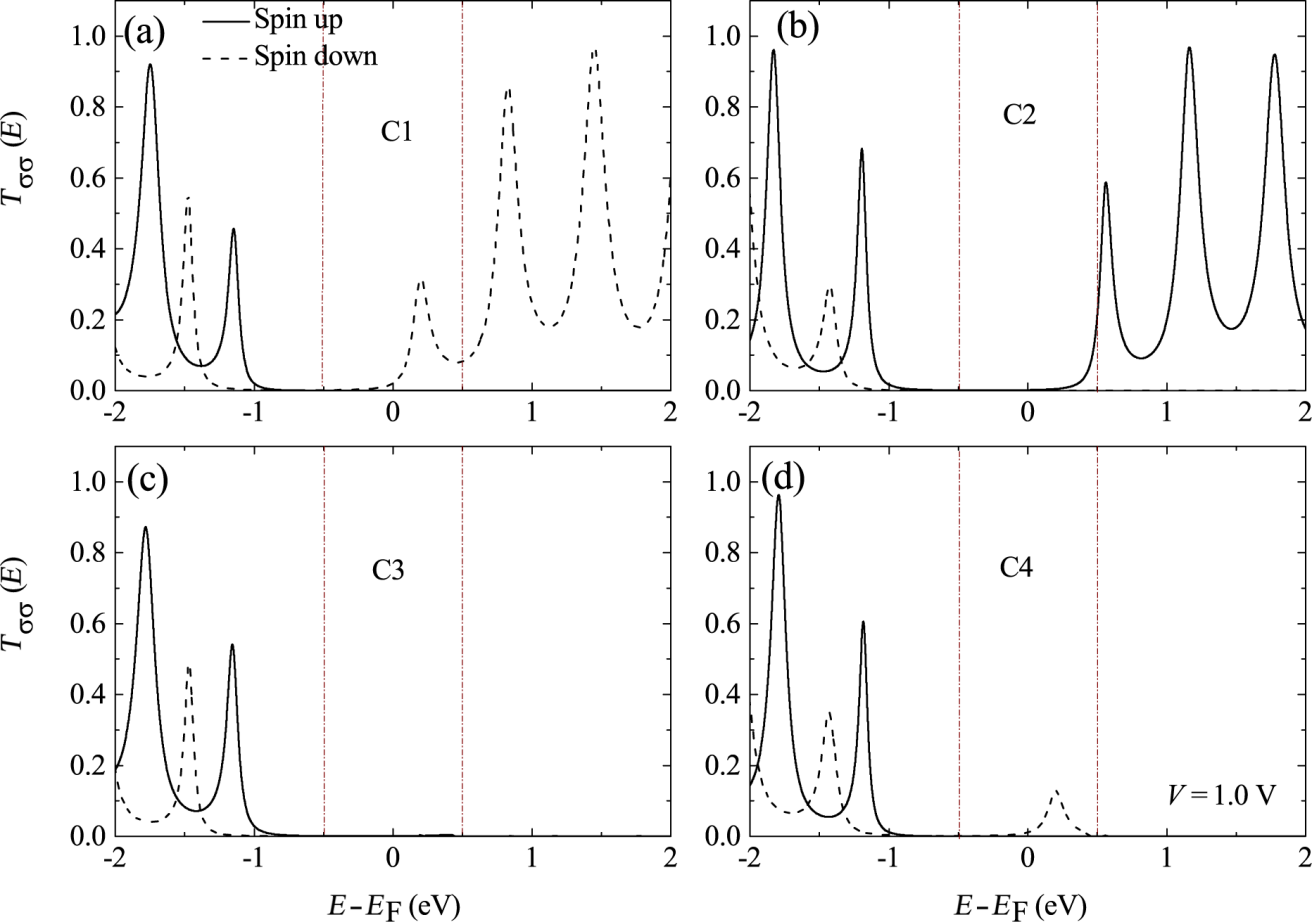
Spin-dependent transmission *T*_σσ_(*E*) as a function of energy close to the Fermi energy for the four magnetization configurations. Panels (a–d) correspond to configurations C1–C4, respectively. The bias voltage is *V* = 1.0 V. Reproduced with permission from [32], copyright 2014 AIP Publishing.

The above analysis may be verified by a calculation of the transmission spectrum. Figure 7 shows the spin-dependent transmission of the four configurations at 1.0 V. For C1 and C4, an efficient transmission peak contributed by the spin-down electrons is found in the bias window, which leads to the higher current shown in Figure 6. However, for C2 and C3, there is no transmission peak in the bias window, and thus a low current is obtained. This is because for the present parameters, the molecular orbital of the OF closest to the Fermi energy is the spin-down LUMO, as shown in Figure 5b. Therefore, only spin-down electrons can tunnel at low bias for C1 and C4. Note that for C2 the tail of the transmission peak from the higher-energy spin-up LUMO enters the bias window, which contributes to the small current seen in Figure 6.

Other designs of four-state resistive devices have also been reported, where a ferroelectric barrier was introduced between two ferromagnets [48–50]. In these designs, both a magnetic field and an electric field are necessary to manipulate both the relative magnetization orientation of the electrodes and the polarization of the barrier. We note that the OF multi-state MR device presented here can be manipulated by only one magnetic field that controls the relative magnetizations. For this, it is useful to employ two ferromagnets with different coercive fields, such as LSMO (30 Oe) and Co (150 Oe) [6]. Then, the different magnetization configurations can be realized by tuning the strength of the magnetic field.

The exploration of OFs in MR devices is still in its infancy except for several pioneering experimental works. For example, Yoo et al. [25] and Li et al. [26] have sandwiched the organic magnet V[TCNE]*_x_* between two Au electrodes or Fe and Al electrodes and demonstrated a room-temperature MR. Organic magnets have also been utilized as spin injectors in organic spin valves. Yoo et al. [51] have constructed V[TCNE]*_x_*/rubrene/LSMO junctions and have observed a MR of about 2.5%. Even all-organic spin valves employing organic magnets as both the spin injector and detector have been designed [52], but only a small negative MR of about 0.04% has been observed. However, based on these proofs of principle and the discussed theoretical progress, the utilization of OFs in MR devices looks promising.

### Spin-current rectification in asymmetric magnetic co-oligomer devices

The aforementioned studies of spin-dependent transport through OF devices are limited to uniform OF molecular chains. In this section, we discuss the electron transport through an asymmetric OF chain, e.g., a magnetic/nonmagnetic co-oligomer, where a spin-current rectification phenomenon can occur. Rectification of the charge current (CC) refers to an asymmetric current–voltage curve under reversal of the bias voltage. Molecular rectification has been proposed and investigated in the past decades, where the spatial asymmetry of the device, either at the molecule/electrode interfaces or in the central molecule, is necessary [36,53–57]. Spin-current rectification describes an asymmetric spin current (SC) upon reversal of the bias, which is more complex than a CC rectification. One may define the CC as *I*_c_ = *I*_↑_ + *I*_↓_ and the SC as 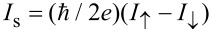. Hence, a SC contains two characteristics: the amplitude of the current and its SP. As a result, the asymmetry of the SC upon bias reversal can have two origins: The first is that the amplitude of the current is not symmetric, while the SP remains unchanged. This is analogous to CC rectification. We call this effect parallel SC rectification. The second is that the SP is reversed upon bias reversal, which we call antiparallel SC rectification. In the following, we review results [33] that demonstrate that both types of SC rectification may be realized in suitably designed OF devices.

**Figure 8:**
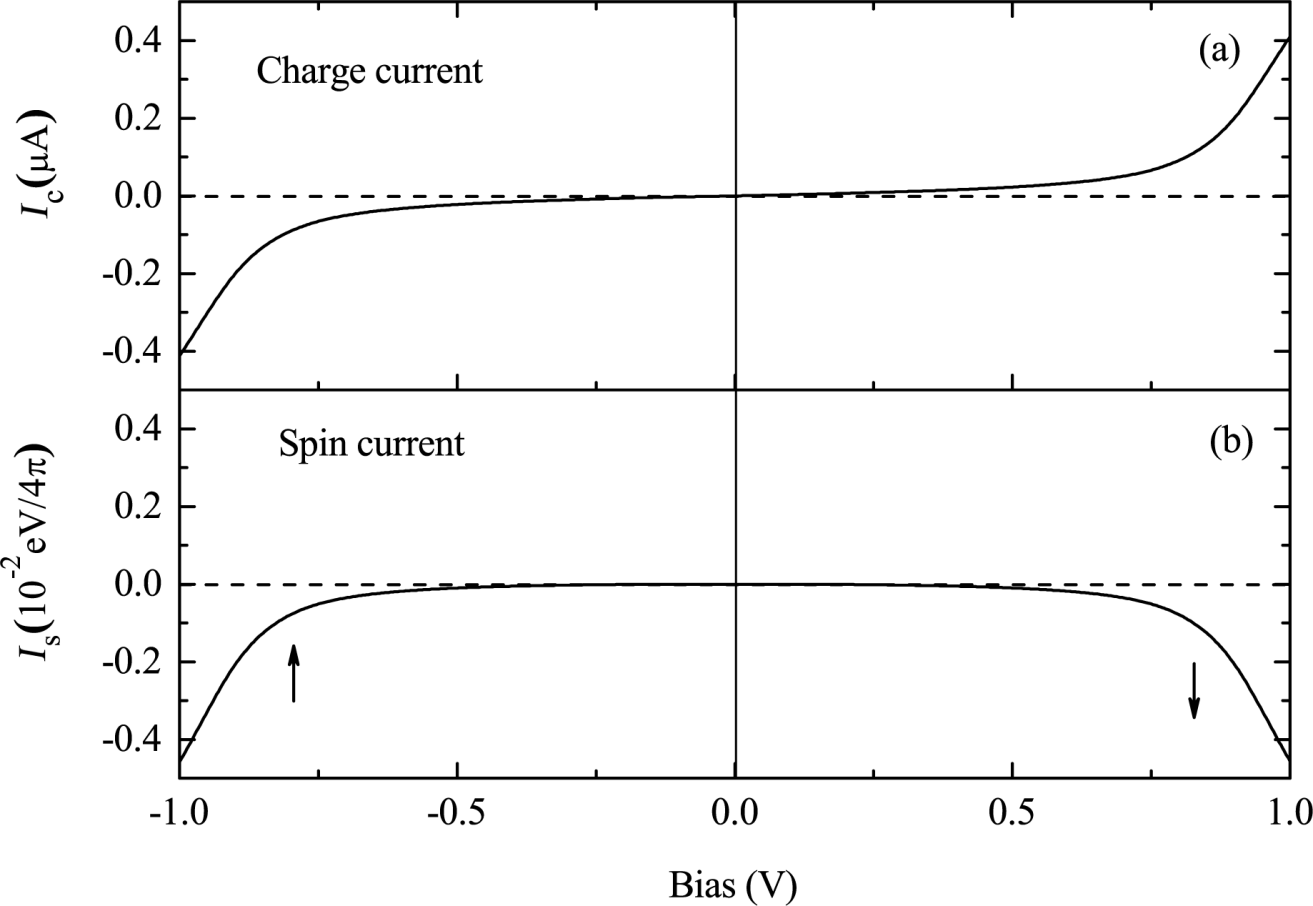
Bias-dependent (a) CC and (b) SC through an asymmetric OF device with *N* = 20 and *E*_F_ = 0. The arrows in (b) indicate the SP of the current. Reproduced with permission from [33], copyright 2008 AIP Publishing.

The OF spin diode consists of a magnetic co-oligomer coupled to two nonmagnetic electrodes [33]. The central magnetic co-oligomer is composed of a left OF molecule and a right nonmagnetic one, such as poly-BIPO and polyacetylene, respectively. The magnetic co-oligomer may be described by the Hamiltonian *H*_OF_ in Equation 1, with the modification that the coupling to radical spins only exists for the odd sites of the left half chain. For simplicity, the electron–electron interaction has been neglected. Results for the CC and SC through the device for the Fermi level in the middle of molecular gap, i.e., *E*_F_ = 0, are shown in Figure 8. In the considered bias region of [−1.0 V,+1.0 V], the CC is symmetric upon bias reversal. The CC begins to increase quickly when the bias approaches ±0.8 V. The magnitude of the current reaches 0.4 μA at ±1.0 V. Rectification of the CC is not observed. However, for the SC, the SP is reversed, although the amplitude of the SC is symmetric. According to the definition of the SC, the current is spin-down polarized for positive bias, whereas it is spin-up polarized for negative bias. This is the antiparallel rectification of the SC defined above.

**Figure 9:**
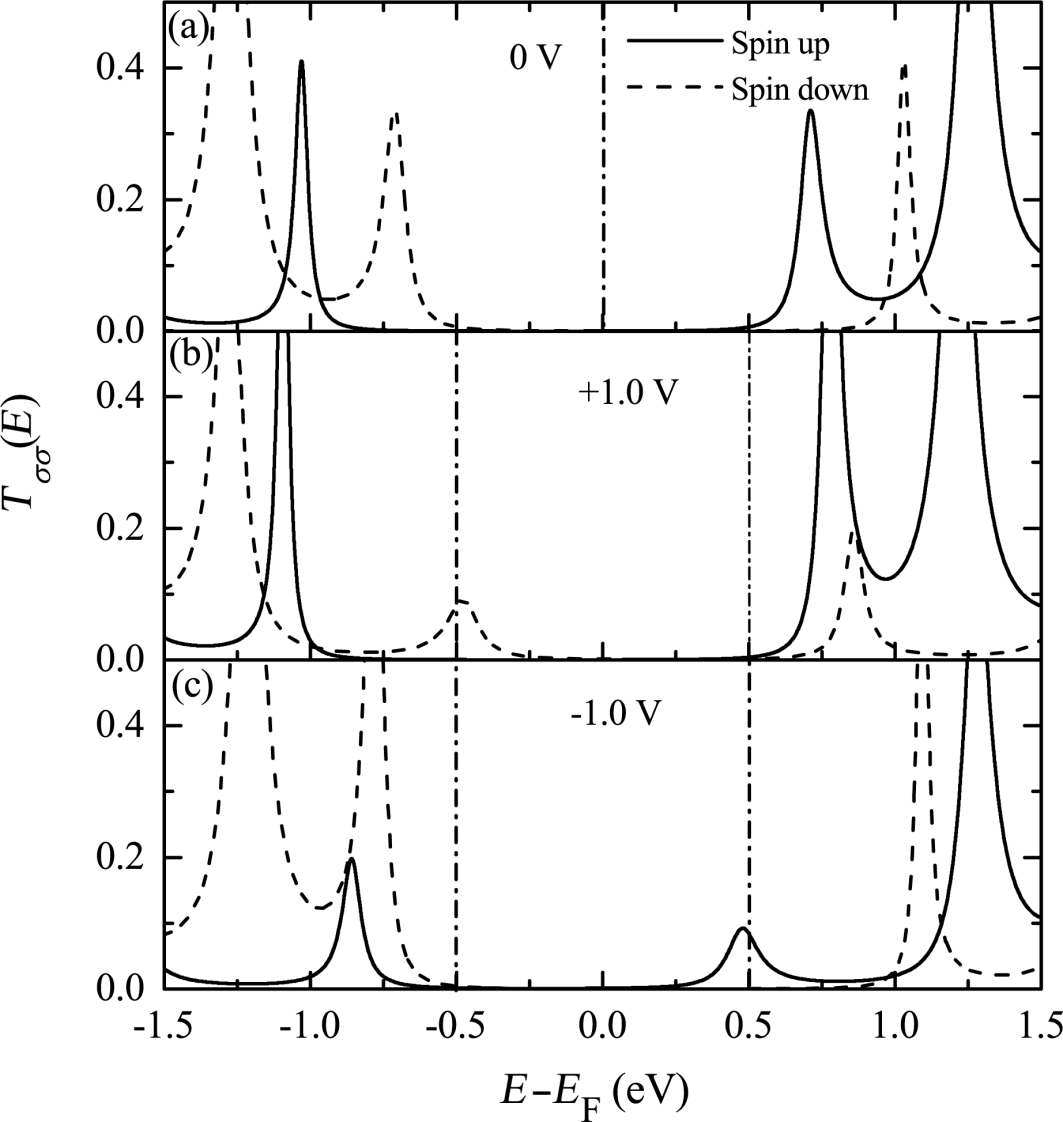
Spin-dependent transmission near the Fermi energy for *N* = 20 and *E*_F_ = 0 at the bias voltages (a) 0 V, (b) +1.0 V, and (c) −1.0 V. Reproduced with permission from [33], copyright 2008 AIP Publishing.

The mechanism of the antiparallel rectification can be explored by investigating the spin-dependent transmission under various biases. In Figure 9, the spin-resolved transmission spectra at 0 V and ±1.0 V are shown. At 0 V, there are two transmission peaks with equal distance from the Fermi energy, which result from the spin-up LUMO and the spin-down HOMO. Applying a positive bias of 1.0 V, the transmission peak of the spin-down HOMO is shifted closer to the Fermi energy and enters the bias window, which contributes a spin-down polarized current. Conversely, when a negative bias of −1.0 V is applied, only the spin-up LUMO peak is in the bias window. One can show that the spin-down LUMO and the spin-up HOMO evolve symmetrically with the bias. As a result, the magnitude of the current is symmetric upon bias reversal but the SP is reversed.

**Figure 10:**
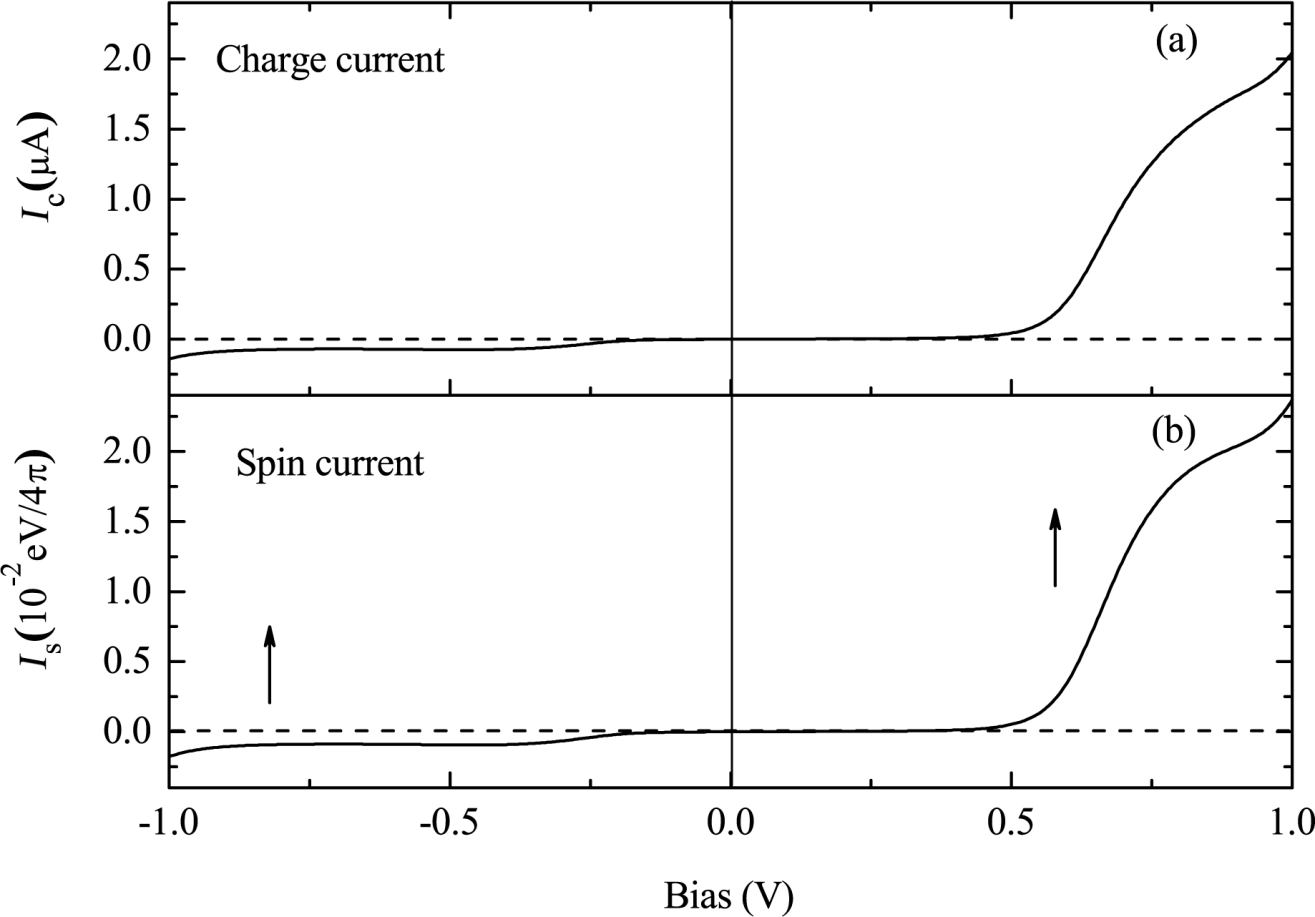
Bias-dependent (a) CC and (b) SC for *N* = 32 and *E*_F_ = 0.3 eV. The arrows in panel (b) indicate the SP of the current. Reproduced with permission from [33], copyright 2008 AIP Publishing.

**Figure 11:**
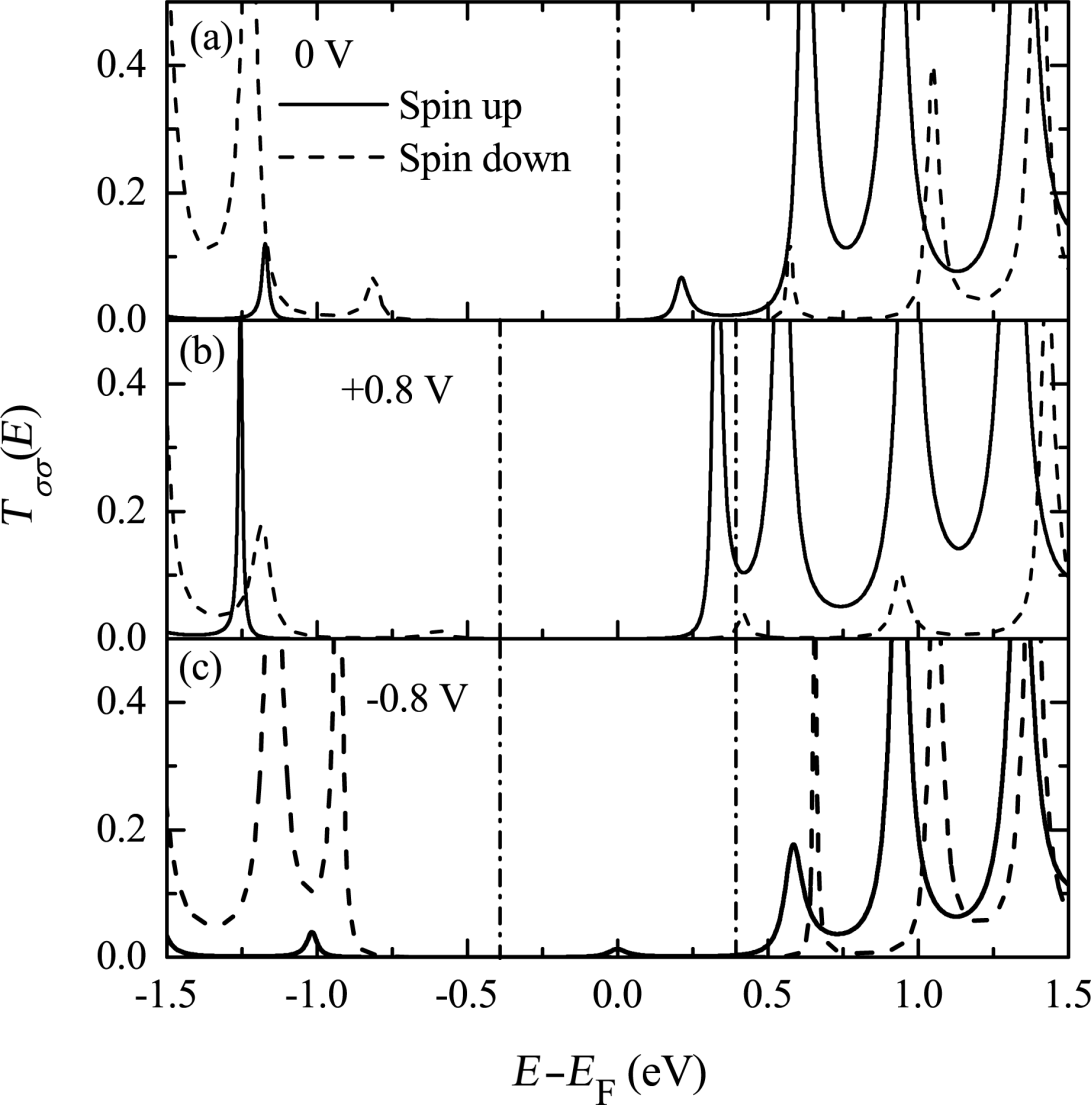
Spin-dependent transmission near the Fermi energy for *N* = 32 and *E*_F_ = 0.3 eV at the bias voltages (a) 0 V, (b) +0.8 V, and (c) −0.8 V. Reproduced with permission from [33], copyright 2008 AIP Publishing.

The relative position of the electrode Fermi level with respect to the molecular energy gap is important for the rectification behavior. If the Fermi level does not lie in the middle of the molecular energy gap, the symmetry of the current contributions from the two nearest peaks is broken. Results for an electrode Fermi level of *E*_F_ = 0.3 eV are shown in Figure 10. Both the CC and SC are rectified with a similar shape of their current–voltage curves. A larger current is obtained for positive bias, where the rectification ratio, defined as RR(*V*) = −*I*_c_(*V*)/*I*_c_(−*V*) for CC and SRR(*V*) = −*I*_c_(*V*)/*I*_c_(−*V*) for SC, reaches about 22 at 0.8 V for both CC and SC. The rectification of SC is of the type of parallel rectification, where only the amplitude of the current is asymmetric and the SP remains unchanged upon bias reversal. The underlying mechanism can be understood from the bias-dependent transmission spectrum, which is shown in Figure 11. In this case, the transmission peak from the spin-up LUMO is mainly responsible for the current under either positive or negative bias. However, the height of the peak is strongly enhanced at positive bias compared to negative bias, leading to a larger spin-up polarized current at positive bias.

**Figure 12:**
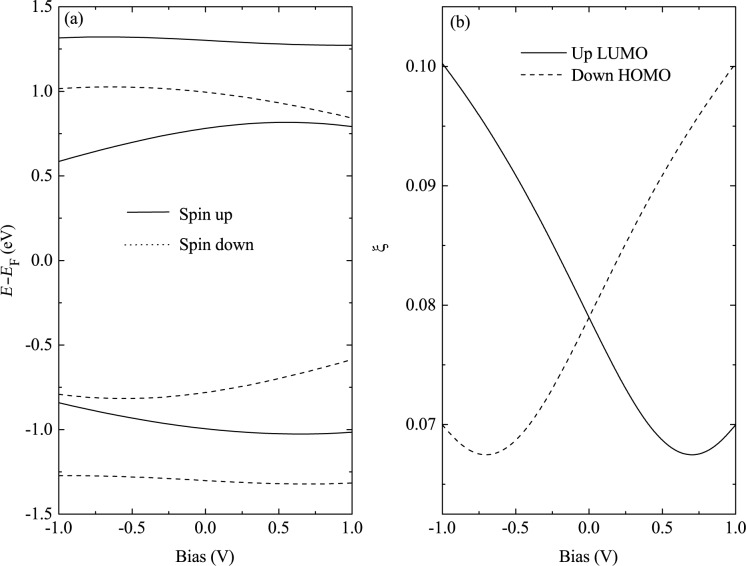
Bias-dependent (a) molecular eigenlevels and (b) electronic localization of the spin-up LUMO and the spin-down HOMO. Reproduced with permission from [59], copyright 2016 Elsevier.

In principle, the asymmetric transport properties of the OF spin diode originates from the asymmetric response of the molecular eigenstates to a bias voltage. Figure 12 shows the bias-dependent molecular eigenlevels and the electronic localization of the two orbitals closest to the Fermi energy, i.e., the spin-up LUMO and the spin-down HOMO. The localization of an eigenstate is defined as 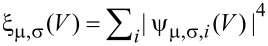
[58], where ψ_μ_*_,_*_σ_*_,i_*(*V*) is the wave function of the molecular eigenstate μ with spin σ at site *i*, for a bias voltage *V*. A larger ξ means a more strongly localized orbital. Figure 12a demonstrates an asymmetric shift of the molecular eigenlevels under positive and negative biases, especially for the orbitals near the Fermi energy. The shift is opposite for the spin-up LUMO and the spin-down HOMO. This shift of the molecular eigenlevels corresponds to the asymmetric shift of transmission peaks shown in Figure 9 and Figure 11. The electronic localization for the spin-up LUMO and the spin-down HOMO is also asymmetric upon bias reversal. For example, the spin-up LUMO tends to be delocalized at positive bias, whereas it becomes more localized at negative bias. A more delocalized orbital will lead to a larger transmission coefficient, which is the reason for the rectification shown in Figure 11.

It should be pointed out that the bias-induced evolution of molecular eigenstates is opposite for the spin-up LUMO and the spin-down HOMO, including the energy shift and the change of electronic localization. If the dominant orbital for the transport is changed, e.g., by using a gate voltage, an interesting phenomenon, namely an inversion of the rectification, may happen. This has been discussed in detail in [59]. Note that the concept of SC rectification reviewed here is based on spin-polarized charge transport. This phenomenon has also been reported in a molecular junction with one ferromagnetic and one nonmagnetic electrode [60]. A distinct scheme of rectification compared to our picture has been proposed recently, which leads to a pure SC, that is, a flow of angular momentum without accompanying CC. This type of SC rectification may be generated by spin pumping techniques [61] or via the spin-Seebeck effect [62].

The above proposed prototype of a molecular spin diode has been supported by ab initio calculations. Zhu et al. [63] have studied a biradical (5-bromo-2,4-dimethoxy-1,3-phenylene)bis(*N*-*tert*-butylnitroxide) molecule connected to two gold electrodes. The molecule is magnetic and spatially asymmetric. The authors have found CC and SC rectification through the device. A high rectification ratio exceeding 100 is reported. Experimental tests of the theoretical predictions are highly desirable.

## Conclusion

In this contribution, theoretical results on spin-dependent electron transport through OFs have been reviewed, focusing on our designs of several OF spintronic devices. They are based on a combination of the extended SSH model and the Green’s function method, including the intrinsic interactions in OFs, i.e., the e–l interaction and the coupling between the spins of π-electrons and radicals. Using the pure OF poly-BIPO as an example, we have discussed the realization of three important concepts for spintronics with OFs: spin filtering, magnetoresistance, and spin-current rectification.

Spin filtering can be realized with metal/OF/metal sandwich structures [31]. An oscillating SP of the current as a function of the bias voltage in predicted for such devices. An extremely large SP is achieved in a certain bias region, which shows that a strong spin-filtering effect is realized. By examining the DOS, it was found that the spin splitting of π-orbitals induced by the coupling between the spins of π-electrons in the main chain and the residual spins of radicals is responsible for the SP, while the large Peierls energy gap induced by the strong e–l interaction is crucial for the nearly complete SP.

Then, a magnetoresistive device based on coupling the OF to two ferromagnetic electrodes [32] has been reviewed. Considering the possible orientations of the magnetization in each component, four distinct magnetic configurations of the device were proposed and the transport in each case were investigated. By calculating the current–voltage characteristics, it was found that the current depends strongly on this configuration and a four-state magnetoresistance was predicted. The intrinsic mechanism was revealed by the transmission analysis, where the spin-resolved electron tunneling between the two ferromagnets suffers a further spin selection by the OF. Using two ferromagnets with different coercive fields as the electrodes should allow one to manipulate a multi-state magnetoresistance device by a magnetic field.

Finally, the additional functionality of spin-current rectification can be implemented by replacing the OF by an asymmetric magnetic co-oligomer, for example consisting of poly-BIPO and polyacetylene [33]. It was found that two types of SC rectification may be realized in such spin diodes by adjusting the position of the Fermi energy of the electrodes relative to the molecular energy levels. For parallel SC rectification, only the amplitude of the SC is asymmetric under reversal of the bias, while the SP remains unchanged. This effect is accompanied by a CC rectification. The other type is antiparallel SC rectification, where only the SP of the current is reversed under reversal of the bias. The origin of the SC rectification can be traced back to the bias-induced asymmetric response of molecular eigenstates, which involves both an asymmetric shift of eigenlevels and an asymmetric localization of orbitals.

We should mention that the reviewed works are limited to the regime of coherent transport in nanoscale devices. A comprehensive study beyond coherent transport is required for the future. Especially for large-scale devices composed of long OF polymer chains, polaronic transport is possible, which is very common in organic materials. One of our works not discussed in detail here exhibits a distinctive property of polarons in OFs caused by the spin radicals, namely spin-charge disparity [64]: The charge and spin distributions of a polaron are shifted with respect to each other. This is expected to lead to novel effects for polaron transport in OFs, which will be investigated with a nonadiabatic dynamics method in the future. Furthermore, the coupling between π-electron and radical spins has so far been treated in a mean-field approximation, which neglects the dynamics of the radical spins. Spin and also charge transport is expected to be affected by the dynamics, which requires a quantum-mechanical description since the radicals typically carry spins *S* = 1/2. Another aspect worth studying is the role of disorder, which is generically important in one-dimensional systems [65]. Finally, a simple spin-independent interfacial coupling between the OFs and the electrodes is considered here. In actual devices, orbital hybridization may happen between the interacting atoms, which will modify the spin states of both the molecules and the metal atoms close to the interface. Ab initio tools will be useful in determining the details. In spite of this, we hope that the works discussed in this contribution deepen our understanding of the electron transport through OFs, and increase the interest in the design of organic spintronic devices with OFs.
